# Bone circuitry and interorgan skeletal crosstalk

**DOI:** 10.7554/eLife.83142

**Published:** 2023-01-19

**Authors:** Mone Zaidi, Se-Min Kim, Mehr Mathew, Funda Korkmaz, Farhath Sultana, Sari Miyashita, Anisa Azatovna Gumerova, Tal Frolinger, Ofer Moldavski, Orly Barak, Anusha Pallapati, Satish Rojekar, John Caminis, Yelena Ginzburg, Vitaly Ryu, Terry F Davies, Daria Lizneva, Clifford J Rosen, Tony Yuen

**Affiliations:** 1 The Mount Sinai Bone Program, Departments of Pharmacological Sciences and of Medicine, and Center of Translational Medicine and Pharmacology, Icahn School of Medicine at Mount Sinai New York United States; 2 Maine Medical Center Research Institute Scarborough United States; Augusta University United States; Augusta University United States

**Keywords:** bone biology, osteoporosis, integrative physiology

## Abstract

The past decade has seen significant advances in our understanding of skeletal homeostasis and the mechanisms that mediate the loss of bone integrity in disease. Recent breakthroughs have arisen mainly from identifying disease-causing mutations and modeling human bone disease in rodents, in essence, highlighting the integrative nature of skeletal physiology. It has become increasingly clear that bone cells, osteoblasts, osteoclasts, and osteocytes, communicate and regulate the fate of each other through RANK/RANKL/OPG, liver X receptors (LXRs), EphirinB2-EphB4 signaling, sphingolipids, and other membrane-associated proteins, such as semaphorins. Mounting evidence also showed that critical developmental pathways, namely, bone morphogenetic protein (BMP), NOTCH, and WNT, interact each other and play an important role in postnatal bone remodeling. The skeleton communicates not only with closely situated organs, such as bone marrow, muscle, and fat, but also with remote vital organs, such as the kidney, liver, and brain. The metabolic effect of bone-derived osteocalcin highlights a possible role of skeleton in energy homeostasis. Furthermore, studies using genetically modified rodent models disrupting the reciprocal relationship with tropic pituitary hormone and effector hormone have unraveled an independent role of pituitary hormone in skeletal remodeling beyond the role of regulating target endocrine glands. The cytokine-mediated skeletal actions and the evidence of local production of certain pituitary hormones by bone marrow-derived cells displays a unique endocrine-immune-skeletal connection. Here, we discuss recently elucidated mechanisms controlling the remodeling of bone, communication of bone cells with cells of other lineages, crosstalk between bone and vital organs, as well as opportunities for treating diseases of the skeleton.

## Introduction

Bone is a highly organized structure consisting of a protein matrix, primarily type 1 collagen, with hydroxyapatite mineral and cells from different lineages interspersed throughout. Skeletal tissue is composed of two distinct micro-skeletal structures—cortical bone (~80%) and trabecular bone (~20%)—that function as sites of muscle and tendon attachment for locomotion, as major storage sites for calcium, phosphate ions required for intergenerational transfer during procreation, and, as has more recently been established, endocrine organs secreting peptides working on other remote organs.

Bone remodeling, a process in which bone resorption is followed by bone formation in a well-defined spatiotemporal sequence, involves the coordinated activity of osteoclasts and osteoblasts, respectively, both of which differentiate from bone marrow precursors that lie in close proximity (Evans, 2007; Zaidi, 2007). Osteoclasts resorb old or damaged bone through the secretion of acid and enzymes that dissolve hydroxyapatite and digest the protein matrix, and their activity is tightly regulated by calcium and hydrogen ion concentrations that they generate locally (Arnett and Dempster, 1986; Zaidi et al., 1989). The resorptive hemivacuole then fills up with osteoblasts of the mesenchymal stem cell origin, which deposits collagen and non-collagenous proteins and that ultimately undergo mineralization through hydroxyapatite deposition. Osteoblasts that get embedded within the bone matrix become osteocytes, the skeletal equivalent of neurons, which sense and respond to mechanical stresses during terrestrial impact using their intertwined dendritic processes traversing the extensive lacunar network within bone (Iqbal and Zaidi, 2005). Osteoblasts, osteoclasts, and osteocytes communicate extensively with each other to couple bone formation and bone resorption. Over the years, it has also become increasingly clear that bone cells in the skeleton intimately interact with immune cells, adipocytes, and hematopoietic cells in the bone marrow, and they are further regulated by the central nervous system, pituitary gland, muscle, and fat.

In all, long-standing efforts to characterize the pathophysiology of aberrant loss or gain of bone in human disease have shed light on novel mechanisms and, importantly, unmasked new actionable targets. Below, we will discuss cellular crosstalk between bone cells, as well as the communication between the skeleton and other organ systems, namely, immune, nervous, neuroendocrine, and other major organs to highlight the therapeutic applications of integrative bone physiology.

## Osteoblast and osteoclast activities are coupled in space and time

Insights into the coupling of bone resorption and bone formation have come to light with the realization that transforming growth factor (TGF) β1 is a central player. TGFβ1, released from bone matrix during resorption, is the primary inducer of bone marrow-derived mesenchymal stem/stromal cell (BMSC) migration to the resorptive hemivacuole as well as their spatial localization (Iqbal et al., 2009; Tang et al., 2009). Disrupting the TGFβ1 gradient in mice with an activating *Tgfb1* mutation recapitulates Camurati-Engelmann disease, characterized by disorganized stromal cell recruitment, dysplastic bones, and increased risk of fracture (Tang et al., 2009).

**Figure 1.**
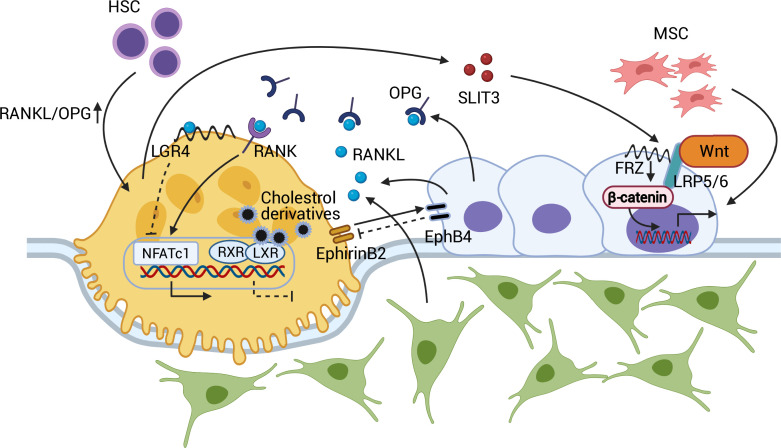
Coupling between osteoblasts and osteoclasts. RANKL/OPG regulates osteoclastogenesis through RANK and downstream NFATc1 activation. RANKL-LGR4 binding creates a negative feedback loop by inhibiting NFATc1. LXR-RXR suppresses osteoclastogenesis upon binding of cellular cholesterol derivatives. EphB4-EphrinB2 interaction promotes osteoblastogenesis and suppresses osteoclastogenesis. SLIT3 from osteoclasts activates WNT/β-catenin and stimulates osteoblast migration and proliferation. Abbreviations: Hematopoietic stem cell (HSC); mesenchymal stem cell (MSC); receptor activator of nuclear factor kappa-Β (RANK); receptor activator of nuclear factor kappa-Β ligand (RANKL); leucine-rich repeat-containing G protein-coupled receptor 4 (LGR4); liver X receptors (LXRs); retinoic acid receptor (RXR); nuclear translocation of nuclear factor of activated T cells cytoplasmic, calcineurin-dependent 1 (NFATc1); slit guidance ligand 3 (SLIT3); osteoprotegerin (OPG); frizzled (Frz).

Parathyroid hormone (PTH) action on the skeleton represents a classical example of osteoblast-osteoclast coupling. Increased osteoclastic bone resorption from continuous PTH exposure is mediated through PTH receptor activation in osteoblasts, not osteoclasts (McSheehy and Chambers, 1986). The activated PTH/PTH receptor stimulates the secretion of receptor activator of nuclear factor kappa-Β ligand (RANKL), a member of the tumor necrosis factor alpha (TNFα) family that binds to RANK on osteoclast precursors to induce osteoclastic differentiation (Hsu et al., 1999; Huang et al., 2004; Lacey et al., 1998; Simonet et al., 1997). RANKL is also secreted by osteocytes, and the osteocyte-selective deletion of *Rankl* results in lower number of osteoclasts, highlighting the role of the osteocyte as a mechanosensor that coordinates site-specific osteoclast recruitment and bone resorption (Nakashima et al., 2011; Xiong et al., 2011; Xiong et al., 2015). Another key player in RANK-RANKL axis that couples osteoblastic activation with osteoclastic resorption is osteoprotegerin (OPG), again secreted by osteoblasts, which serves as a decoy receptor to RANKL and regulates RANK/RANKL binding ratio, and consequently, the rate of osteoclasts differentiation and action (Simonet et al., 1997; Figure 1).

The recent identification of liver X receptors (LXRs) has provided further insight into the endogenous mechanism that determines the RANKL/OPG equilibrium within osteoblasts (Kleyer et al., 2012). The two types of LXRs, α and β, have been known to regulate cholesterol metabolism and the immune response (Zelcer and Tontonoz, 2006). However, in co-cultures of osteoblasts and osteoclasts, LXR ligand treatment decreased the RANKL/OPG ratio and interfered with osteoblast-induced osteoclastogenesis. This in vitro finding was supported by an ovariectomized rodent model in which LXR agonist administration attenuated osteoclast differentiation and rescued ovariectomy-induced bone loss (Kleyer et al., 2012). In this context, leucine-rich repeat-containing G protein-coupled receptor 4 (LGR4) also regulates osteoclast differentiation. LGR4, expressed in osteoclasts, binds RANKL and prevents the nuclear translocation of nuclear factor of activated T cells, cytoplasmic, calcineurin-dependent 1 (NFATc1), preventing osteoclastogenic gene expression. Moreover, activated NFATc1 induces transcription of *Lgr4* in osteoclasts, negatively regulating RANK/RANKL-induced osteoclastogenesis (Luo et al., 2016; Zaidi and Iqbal, 2016; Figure 1).

Osteoblast-osteoclasts coupling is in fact bidirectional in that osteoclasts also affect osteoblast formation and function. EphrinB2, a transmembrane protein expressed by osteoclasts, interacts with its receptor, EphB4, on osteoblasts. EphirinB2-EphB4 signaling in osteoclasts suppresses osteoclastogenesis by inhibiting the c-Fos-NFATc1 signal, whereas in osteoblasts, it upregulates osteogenic genes, such as *Osx* and *Runx2*, and promotes bone formation (Zhao et al., 2006). Further, the osteoclast-secreted axon guidance molecule, SLIT3, stimulates osteoblast migration and proliferation by activating β-catenin. Osteoclast-specific *Slit3*-deficient mice thus demonstrate reduced bone mass, whereas osteoblast-specific *Slit3* deletion results in normal bone mass (Kim et al., 2018; Figure 1).

Sphingolipids and other membrane-associated proteins also facilitate synchronous osteoblast-osteoclast coupling. Sphingosine-1-phosphate, a signaling sphingolipid, controls both osteoblastic bone formation and osteoclast precursor migration during bone remodeling. It also functions as a chemoattractant, directing osteoclast precursors to sites of stress (Ishii et al., 2009; Pederson et al., 2008). Likewise, semaphorins, a class of membrane-associated secreted proteins, either enhance or suppress bone formation. Semaphorin 3A (*Sema3A*)-deficient mice receiving recombinant Sema3A display a rescue of osteoblastic bone formation and osteoclastic bone resorption (Hayashi et al., 2012; Zaidi and Iqbal, 2012). Osteoclast-derived Sema4D, however, suppresses bone formation when it binds to its osteoblast receptor Plexin-B1 (Negishi-Koga et al., 2011).

In all, understanding the coupling of bone cells has prompted new treatments for bone diseases, such as osteoporosis, and continue to offer potential therapeutic targets. A monoclonal antibody against RANKL, denosumab, is currently popular for treating both osteoporosis and skeletal metastasis (McClung et al., 2006). Given that targeting LGR4 only affects mature osteoclasts and not precursor cells, there is a potential in using its extracellular domain as a means of binding excess RANKL to restrict bone resorption (Luo et al., 2016; Zaidi and Iqbal, 2016). Furthermore, enhancing EphrinB2-EphB4 or inhibiting Sema4D holds therapeutic promise to promote bone formation.

## Developmental genes reawaken during adult bone remodeling

Three critical developmental pathways, namely bone morphogenetic protein (BMP), NOTCH, and WNT signaling pathways, remain active beyond morphogenesis to regulate adult bone remodeling. They interact with the master transcriptional regulators RUNX2*,* Osterix (OSX), activating transcription factor 4 (ATF4), and Schnurri-2. Other developmental genes that promote pluripotency, such as octamer-binding transcription factor 4 (*Oct4*) and sex-determining region Y*-*box 2, are also expressed in mesenchymal stem cells during osteogenic differentiation (Matic et al., 2016a). However, it has been proven conclusively that *Oct4* has no role in bone homeostasis.

A fundamental role for members of the BMP family in skeletal development and remodeling is well documented. Osteoblasts and osteoclasts express multiple BMPs, namely (BMP-2, -4, -5, -7, and -9) and the BMP receptors (BMPRs), type I and II (Huntley et al., 2019; Wu et al., 2016). Intracellular BMP signaling is mediated by SMAD-dependent and non-SMAD-dependent pathways. BMP/BMPR binding phosphorylates the BMP-specific receptor-regulated SMADs, SMAD-1, -5, and -8 to form a heterodimeric SMAD-1/5/8 complex that translocates to the nucleus with SMAD-4. In the non-SMAD-dependent pathway, BMP/BMPR binding phosphorylates TGFβ-activated kinase (TAK1) and activates the JNK and p38 MAPK signaling pathways. Both pathways then increase the transcriptional activity of *Runx2*, *Dlx5*, and *Osx* (Wan and Cao, 2005; Wu et al., 2016). The BMP signaling pathway is regulated at many levels. Noggin (NOG), a glycoprotein secreted by osteoblasts, binds BMPs selectively and competitively inhibits BMP action on the cell surface. Osteoblast-specific *Nog* overexpression in mice shows decreased trabecular bone volume and impaired osteoblast function with increased fractures (Devlin et al., 2003). NOG levels in mice appear to increase with aging, which might contribute to age-related low bone turnover (Wu et al., 2003). Inhibitory SMAD proteins, such as SMAD-6 and -7, prevent downstream phosphorylation of the SMAD-1/5/8 complex. The BMP pathway is also regulated by the ubiquitin-proteasome system. For example, the SMAD-specific E3 ubiquitin protein ligase (Smurf)-1 downregulates BMP signaling in osteoblasts by promoting SMAD-1 degradation (Zhao et al., 2004; Zhu et al., 1999). Smurf1 also mediates TNF-induced suppression in osteoblastogenesis through its interaction with SMAD-6 and RUNX2 downregulation (Kaneki et al., 2006; Shen et al., 2006). Lastly, the ubiquitin-conjugating enzyme 9 targets SMAD-4 for degradation to suppress BMP pathway signaling (Wan and Cao, 2005).

The NOTCH receptor, a single transmembrane domain receptor protein required for somite maturation, is another critical developmental molecule that also regulates postnatal skeletal homeostasis. The binding of its ligands, JAGGED-1 and -2 and delta-like ligand 1–3, results in the cleavage of the NOTCH intracellular domain (NICD) by γ-secretases presenilin-1 and -2 (Bassett et al., 2008). The cleaved, active NICD undergoes nuclear translocation and interacts with CSL (CBF1, suppressor of hairless, lag-1) transcription factors to activate target gene expression (Luo et al., 2019). NOTCH signaling triggers proliferation and maintains a pool of osteoblast progenitors, while repressing differentiation of early osteoblasts to terminally differentiated cells (Engin et al., 2008; Hilton et al., 2008). Up- or downregulated NOTCH signaling yields distinct skeletal phenotypes depending on the stage of osteoblast lineage differentiation. Mice that overexpress *Notch1* driven by an early promoter Col3.6 repress osteoblast differentiation and develop osteopenia (Zanotti et al., 2008). Similarly, patients with Hajdu-Cheney syndrome, a rare genetic disease characterized by significant bone loss and fractures, have gain-of-function *NOTCH2* mutations. This condition was recapitulated in mice with a *Notch2*^Q2319X^ mutation, exhibiting osteopenia with excessive bone remodeling (Canalis et al., 2016). Conversely, when NOTCH ligand JAGGED-1 was deleted in osteoprogenitor cells, increased trabecular bone mass with increased osteoblast activity was noted (Lawal et al., 2017). Likewise, the loss of γ-secretases presenilin-1 and -2 in osteoblast progenitors yielded a high bone mass phenotype at an early age, but the mice progressively lost bone with aging (Engin et al., 2008)—together suggesting that a NOTCH-mediated regulatory loop maintains the population of osteolineage cells.

The WNT signaling pathway is integral to the developmental patterning of the dorsal somite and, being ubiquitously expressed, regulates cell growth and differentiation (Fuentealba et al., 2007). Canonical WNT signaling is initiated upon simultaneous binding of WNT ligands to the frizzled (FRZ) and low-density lipoprotein receptor-related protein (LRP) 5/6 receptors. Activation of the co-receptors leads to the inhibition of glycogen synthase kinase 3 activity and stabilization of β-catenin. The stabilized β-catenin subsequently undergoes nuclear translocation and interacts with the transcription factors T-cell factor and lymphoid enhancer factor to promote osteoblast gene expression (MacDonald et al., 2009). The β-catenin-mediated canonical pathway interacts with the BMP pathway through Axin-related protein (Axin)2, which promotes β-catenin degradation. Axin2-deficient mice display increased bone mass; this anabolic effect is dependent on BMP-2/4 and OSX (Yan et al., 2009).

Murine genome-wide association studies (GWAS) and the identification of WNT gene variants with significant skeletal phenotype, notably *LRP5* mutations causing osteoporosis-pseudoglioma syndrome and *SOST* mutations leading to sclerosteosis and Van Buchem disease, have together established WNT signaling as a cornerstone of skeletal homeostasis (Boyden et al., 2002; Krishnan et al., 2006; Rivadeneira et al., 2009). Mechanistically, WNT/β-catenin activation in adult mice increases bone mass by enhancing stem cell renewal, pre-osteoblast proliferation, and osteoblast differentiation, while inhibiting osteoblast and osteocyte apoptosis (Baliram et al., 2011; Krishnan et al., 2006; Zhang et al., 2013). The downstream effects of WNT signaling, along with β-catenin-mediated osteoclast inhibition, have led to the recent development of an anti-osteoporosis drug with dual pro-anabolic and anti-resorptive actions. Romosozumab, a monoclonal antibody against the bone-specific WNT inhibitor sclerostin, now FDA-approved, has shown promising efficacy in reducing vertebral, non-vertebral, and hip fractures (Cosman et al., 2016; Saag et al., 2017). Dickkopf-1 (DKK1), another WNT inhibitor, contributes to myeloma-related bone disease as the production of DKK1 by myeloma cells increases the RANKL/OPG ratio (Qiang et al., 2008). An anti-DKK1 monoclonal antibody is now being studied for use as a potential therapeutic agent (Fulciniti et al., 2009).

## Chatter between bone and immune cells in bone marrow

The physical proximity of bone and bone marrow allows close interactions between bone cells and bone marrow-derived cells. Osteoclasts, derived from hematopoietic stem cells (HSCs), bear the immune receptor osteoclast-associated receptor (OSCAR) to activate receptor expressed on myeloid cells (TREM) 2, signal-regulator protein beta (SIRPβ) 1, and paired immunoglobulin-like receptor A (PIRA), establishing the physiologic relevance of the osteo-immune interface (Barrow et al., 2011; Otero et al., 2012; Pfeilschifter et al., 1989; Takayanagi, 2007). The RANK-RANKL interaction is mediated through the recruitment of TNF receptor-associated factor 6 and, at the same time, the phosphorylation of immune-receptor tyrosine-based activation motifs (ITAMs), such as DAP12 and Fc receptor subunit, resulting in NF-κB activation and cytosolic Ca^2+^ release. NFATc1 is then activated by calcineurin and amplified in cooperation with activator protein 1 (Asagiri et al., 2005). Consequently, gain-of-function mutations of calcineurin result in markedly increased NFATc1 and osteoclast differentiation, whereas its downregulation suppresses osteoclast formation (Sun et al., 2007). Calcineurin inhibitors like tacrolimus, a commonly used immunosuppressant, can cause low bone turnover and reduced bone formation (Epstein et al., 2003; Sun et al., 2005). *CanA*-deficient mice showed markedly reduced mineral apposition rates with osteogenic genes downregulation, namely, *Runx2*, *Bsp,* and *Ocn* (Sun et al., 2005). In contrast, ITAM-deficient mice (*Dap12^-/-^FcRγ^-/-^) preserve bone mass after ovariectomy* (Wu et al., 2007).

Other immune cells in the bone marrow produce several pro- and anti-osteoclastogenic cytokines that together optimize overall osteoclast differentiation. While TNFα stimulates osteoclastogenesis, IFNγ and interferon regulating factor 8 (IRF8) suppress osteoclast formation. *Irf8*-deficient mice thus show significant osteoporosis due to increased osteoclastogenesis (Zhao et al., 2009). Furthermore, T-helper 17 (Th17) cells secrete predominantly RANKL and TNF (compared with IFNγ), which support its contribution to hyper-resorption in autoimmune arthritis (Komatsu et al., 2014). Th17 cells secrete IL-17A, which is required for PTH to exert its catabolic effects on bone (Li et al., 2015). This is, in part, through the indirect stimulation of RANKL production by osteocytes through IL-17A signaling (Li et al., 2019). Taken together, it is clear that bone and bone marrow-derived cells interact closely to maintain skeletal homeostasis with the immune system serving as a bridge.

## Osteogenesis, hematopoiesis, and angiogenesis in local partnership

Findings over the past decade have established that bone remodeling and blood formation are critically entwined, with the osteoblast playing a central role in the regulation of hematopoiesis. The regulatory microenvironment, or the so-called ‘niche’ where HSCs reside, also involves BMSCs and osteoblasts (Méndez-Ferrer et al., 2010). Spindle-shaped N-cadherin+ CD45 osteoblastic cells and angiopoietin-1-expressing osteoblasts have been shown to protect and maintain HSCs in the niche (Arai et al., 2004; Zhang et al., 2003). Through WNT and NOTCH signaling, they promote HSC renewal, maturation, and survival in response to PTH (Calvi et al., 2003; Fleming et al., 2008; Weber and Calvi, 2010). Other transcriptional factors, like growth factor independence 1b, are also involved in maintaining HSC cellularity and functional integrity through the regulation of WNT signaling (Shooshtarizadeh et al., 2019). This interaction of bone marrow with bone is, in part, mediated by the sympathetic nervous system (SNS). CXCL12, a chemokine that directly causes HSC migration, is regulated by circadian secretion of noradrenaline by sympathetic nerves innervating BMSCs through β-adrenergic receptors (Katayama et al., 2006; Méndez-Ferrer et al., 2008). Thus, disrupting sympathetic signal interferes with HSC maintenance. For example, acute myelogenous leukemia-induced sympathetic neuropathy commits mesenchymal progenitors to the osteoblast lineage at the expense of HSC-maintaining periarteriolar niche cells (Hanoun et al., 2014).

In addition to regulating hematopoiesis, osteoblasts also regulate erythropoiesis by producing erythropoietin (EPO) in response to hypoxia. This increased EPO production is caused by enhanced hypoxia-inducible factor-alpha (HIF-α) signaling in osteoblastic precursors within the niche, resulting in selective expansion of the erythroid lineage (Rankin et al., 2012). Increased EPO and abnormal erythroid proliferation are proposed mechanism of low bone mass in patients with ineffective erythropoiesis such as β-thalassemia. Iron metabolism also plays a significant role in bone remodeling through action of erythroferrone (ERFE), a protein secreted by erythroblasts in bone marrow, a negative regulator of hepcidin. ERFE was shown to bind to BMP family-2, -6, and -2/6 heterodimer (Castro-Mollo et al., 2021; Wang et al., 2020). By using *Erfe*^-/-^ mice and β-thalassemic mice with systemic loss of ERFE expression (*Hbb^th^*^3/+^;*Erfe*^-/-^), our group showed that ERFE was highly expressed in osteoblasts even compared with erythroblasts, and the absence of ERFE caused high bone turnover and significant bone loss. By suppressing bone turnover from downregulating BMP-2-mediated signaling and RANKL production, ERFE was shown to exert bone protective effects (Castro-Mollo et al., 2021).

Augmented HIF-α activity enhances angiogenesis and osteogenesis (Schipani et al., 2009; Wang et al., 2007; Wu et al., 2015). Increased *Hifα* expression results in proliferation of a specific sub-population of endothelial cells (Type H), especially in the metaphysis of long bones, and increases the survival and proliferation of osteoprogenitors (Wang et al., 2007). The coupling of angiogenesis and osteogenesis is further highlighted by the dual role of vascular endothelial growth factor (VEGF)-A. VEGF not only promotes endothelial cell migration and proliferation, but also stimulates osteogenesis through the positive regulation of osteogenic growth factors (Schipani et al., 2009). Molecular crosstalk through angiocrine-, NOTCH-, and NOG-related signals also links angiogenesis and osteogenesis (Kusumbe et al., 2014). Defective angiocrine release of NOG, promoted by NOTCH, results in skeletal defects and impaired angiogenesis (Kusumbe et al., 2014). The effects of these signaling pathways are mediated by a subtype of vessels, called CD31^hi^/Emcn^hi^, which generates a distinct TGFβ3-rich microenvironment to maintain perivascular osteoprogenitors (Kusumbe et al., 2014). All of this together supports the fact that osteogenesis and angiogenesis are coupled tightly, new information that is especially critical in relation to fracture healing (Schipani et al., 2009; Wang et al., 2007).

## Pituitary hormones expand their circuitry

**Figure 2.**
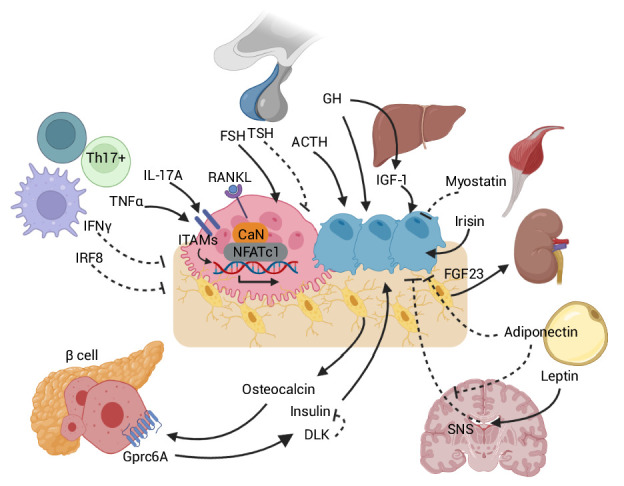
Skeletal crosstalk with other organs. Pituitary hormones directly regulate bone remodeling. FSH stimulates osteoclastogenesis, whereas TSH inhibits osteoclastic bone resorption. ACTH promotes osteoblastic bone formation. GH triggers anabolic signals directly and indirectly through IGF-1. Leptin-mediated SNS activation negatively regulates bone remodeling. The inhibitory peripheral action of adiponectin on bone opposes its centrally mediated action by blocking SNS. OCN, upon binding to the GPRc6A receptor on pancreatic β-cells, can enhance β-cell proliferation and insulin secretion. Insulin binding on osteoblasts can, in turn, promote OCN production. DLK from β-cells counteracts OCN activity by inhibiting the stimulatory effect of insulin. Osteocytes release FGF23, which promotes renal phosphate excretion. Myokines, such as myostatin and irisin, also directly affect bone remodeling. Immune-bone interactions notably occur through various cytokines, such as TNFα, IL-17A, IFNγ, and IRF8. Abbreviations: Follicle stimulating hormone (FSH), thyroid stimulating hormone (TSH), growth hormone (GH), adrenocorticotropic hormone (ACTH), receptor activator of nuclear factor kappa-Β (RANK), sympathetic nervous system (SNS), delta-like protein (DLK), interferon regulatory factor 8 (IRF8), interferon (IFN), tumor necrosis factor (TNF), interleukin (IL), immunoreceptor tyrosine-based activation motif (ITAM), calcineurin (CaN), G protein-coupled receptor class C group 6 member A (GPRC6A), osteocalcin (OCN), fibroblast growth factor (FGF).

The pituitary gland largely orchestrates peripheral hormone secretion from various endocrine organs in response to hypothalamic signals. Beyond their traditional roles, now we know that pituitary hormones also have direct effects on skeleton remodeling (Figure 2). Both osteoclasts and osteoblasts express G protein-coupled receptors (GPCRs) for thyroid stimulating hormone (TSH), follicle stimulating hormone (FSH), growth hormone (GH), adrenocorticotropic hormone (ACTH), prolactin (PRL), oxytocin (OXT), and vasopressin (AVP) (Abe et al., 2003; Fritton et al., 2010; Seriwatanachai et al., 2008; Sun et al., 2006; Tamma et al., 2009; Tamma et al., 2013; Zaidi et al., 2010).

It is challenging to examine an independent skeletal effect of pituitary hormones due to reciprocal relationship between the pituitary and the endocrine targets. Abe et al. first described an independent effect of TSH signaling using haploinsufficient TSH receptor mice (*Tshr^+/-^*) that had normally developed thyroid follicles and normal thyroid function (Abe et al., 2003). Furthermore, induction of an iatrogenic hyperthyroid state in mice supplementing T4 caused profoundly higher bone loss in mice without the TSHR compared with wild type mice (Baliram et al., 2012). This preclinical data is consistent with strong negative correlations between low serum TSH levels and bone turnover markers, bone mineral density and fracture risk from several population-based observational studies (Aubert et al., 2017; Kim et al., 2006; Kim et al., 2021; Morris, 2007).

The skeletal effect of TSH, at least in part, is mediated through bone-active cytokines. TNFα was upregulated In *Tshr*^-/-^ mice, with RANK-L and M-CSF being unchanged (Abe et al., 2003). Compound mutants of TSHR and TNFα deficiency confirmed that TNFα plays a critical role in bone loss and increased osteoclastogenesis in the absence of TSH signaling (Hase et al., 2006; Sun et al., 2013). This is another fascinating example of interaction between endocrine and immune cells on skeletal remodeling (see above). However, the effect of TSH on osteoblasts seems not as straightforward. Our initial description of an anti-osteoblastic effect of TSH in vitro through the downregulation of VEGF receptor (FLK-1) and the WNT co-receptor, LRP5 (Abe et al., 2003), was followed by our intervention study using recombinant human TSH (rhTSH)—this showed clear evidence of an anabolic action in vivo. Intermittent administration of small dose of rhTSH increased osteoblastogenesis and bone mass, without altering thyroid hormones (Sampath et al., 2007; Sun et al., 2008). A direct anabolic effect of rhTSH in terms of inducing an elevation in the bone formation marker procollagen 1 intact N-terminal pro-peptide was also established in people (Martini et al., 2008). Furthermore, subjects with the gain-of-function polymorphism (*TSHR^D727E^*) displayed higher bone mass (Heemstra et al., 2008; van der Deure et al., 2008). Recent findings suggest that TSH-induced osteoblastic action might be mediated by β-arrestin, which serves as a scaffold linking GPCRs to Erk1/2 signaling. TSH-induced binding of β-arrestin-1 to TSHR, which then phosphorylated Akt1, p38, and Erk1/2 and, by doing so, upregulated *Alp*, *Rankl,* and *Opn*. Knockdown of β-arrestin-1 inhibited TSHR-mediated osteogenic gene upregulation (Boutin et al., 2020; Cassier et al., 2017; Ramajayam et al., 2012). In all, these studies are meaningful clinically, in that they could explain the bone loss in patients with subclinical hyperthyroidism, where TSH levels are low and serum thyroid hormones are relatively normal. With that said, it also seems clear that it may be unnecessary to over-suppress serum TSH in patients other than in patients with thyroid cancer, where such suppression is clinically necessary. In that situation, an anti-resorptive therapy may be mandated to prevent bone loss.

In 2006, we reported for the first time that FSH also directly regulates bone remodeling. It acts on FSH receptors (FSHRs) coupled to the G protein, G_i2α_, to increase osteoclastic bone resorption and suppress bone formation (Robinson et al., 2010; Sun et al., 2006; Sun et al., 2007; Zhu et al., 2012b). FSH also enhances RANK and, by doing so, indirectly promotes osteoclastogenesis by stimulating the release or altering the receptor expression of TNFα, IL-1β, and IL-6 (Cannon et al., 2010; Cannon et al., 2011; Iqbal et al., 2006; Wang et al., 2015).

Haploinsufficient FSHR mice (*Fshr*^+/-^) had normal estrogen levels and developed an intact uterus; yet they showed higher bone mass compared with wild type mice (Sun et al., 2006). A separate study also showed higher bone volume and less trabecular spacing in the absence of Fshβ (Morgan et al., 2022). In addition, administering recombinant FSHβ augmented ovariectomy-induced bone loss, while blocking with anti-FSHβ antibody reversed the ovariectomized bone loss (Liu et al., 2010; Zhu et al., 2012b). These findings are underscored by findings from human observational studies using large epidemiologic cohorts of different ethnicity, namely the Study of Women’s Health Across the Nation (SWAN), AGES-Reykjavik Study of Older Adults and Chinese cohorts, all of which noted a strong inverse correlation between serum FSH levels and bone mass independently of estrogen levels (Adami et al., 2008; Cheung et al., 2011; Gallagher et al., 2010; Randolph et al., 2003; Sowers et al., 2003; Veldhuis-Vlug et al., 2021; Wu et al., 2010; Xu et al., 2009). Moreover, women with an activating *FSHR* polymorphism (rs6166) display a lower bone mass and high resorption markers (Rendina et al., 2010).

Taken together, it is plausible that elevated FSH levels, which precede estrogen deficiency during the menopausal transition, contribute to the rapid bone loss that begins during the late perimenopause. Noting the therapeutic relevance of these findings in relation to results from SWAN, we developed a humanized, epitope-specific FSH-blocking antibody as a potential therapeutic for osteoporosis (Gera et al., 2020). Interestingly, we also showed that blocking FSH reduces body fat, increases energy expenditure, and prevents neurodegeneration in mouse models. It is possible therefore that FSH blockade in the early years of the menopause may, in fact, reduce the extent of bone loss, visceral obesity, energy dysregulation, and the spikes of cognitive decline that are noted as early as the late perimenopause, when, as stated above, serum FSH levels are rising in the face of normal estrogen as a response to declining ovarian reserve.

GH is, expectedly, an important hormone as a growth signal that mediates postnatal longitudinal bone growth. It is now clear that GH not only works through IGF-1, but also acts directly on the skeleton (Bouillon, 1991). GH receptor-deficient and IGF-1-deficient mice both showed a similar ~25–30% reduction in body length compared to wild type mice with a significant further reduction in mutant mice lacking both molecules (Lupu et al., 2001). Furthermore, peripheral GH administration enhanced cartilage growth in hypophysectomized and GH-deficient rodents (Isaksson et al., 1987; Isaksson et al., 1991; Ohlsson et al., 1998) and reversed the osteopenic phenotype of estrogen-deficient and liver-derived IGF-1-deficient mice (Fritton et al., 2010)—altogether suggesting a direct local skeletal effect of GH.

The systemic and local effects of IGF-1 on the skeleton have been carefully examined using genetically modified mice. Liver-specific IGF-1-deficient mice, which displayed decreased circulating IGF-1 (by ~75%), surprisingly showed normal skeletal growth with *albeit* impaired cortical bone parameters (Sjögren et al., 1999; Yakar et al., 1999). However, bone-specific IGF-1-deficient mice showed significantly reduced bone size and bone mass, impaired bone formation and mineralization, despite normal levels of circulating IGF-1 (Govoni et al., 2007). This indicates a critical role for locally produced IGF in skeletal regulation. A certain level of systemic IGF-1 is still required for skeletal growth. Systemic IGF-1 deletion caused greater bone loss compared with bone-specific IGF-1 deletion, and the re-expression of liver-specific IGF1 in global IGF-1-deficient mice achieved ~30% of postnatal growth (Stratikopoulos et al., 2008). In addition, a further decrement of IGF-1 below ~10% in liver-specific IGF-1-deficient mice by deleting IGF-binding protein-3 and the acid labile subunit resulted in marked growth retardation (Ohlsson et al., 2009; Yakar et al., 2009). Together, these findings suggest that GH, systemic IGF-1, and local IGF-1 exert a direct effect on postnatal skeletal growth.

We found that ACTH was also directly involved in bone remodeling by bypassing known glucocorticoid-mediated action. ACTH enhances osteoblastic differentiation by upregulating the protease inhibitor alpha-2-macroglobulin, which likely promotes osteoblastic differentiation through TGFβ induction (Sadeghi et al., 2020). It also increases VEGF expression through the melanocortin receptor MC2R on osteoblasts (Zaidi et al., 2010). Given the pathophysiologic role of vascular insufficiency due to VEGF suppression in avascular necrosis (AVN) of the femur, ACTH can be a therapeutic target for treating AVN of the femur (Kerachian et al., 2010; Sadeghi et al., 2020).

Other pituitary hormones like PRL and OXT are also implicated in calcium homeostasis and bone remodeling. Pregnancy and lactation are characterized by excessive maternal bone resorption and bone loss, both of which are reversed upon weaning (Sowers et al., 1995; Wysolmerski, 2002). PRL inhibits bone formation and stimulates bone resorption by suppressing OPG (Coss et al., 2000; Seriwatanachai et al., 2008). During pregnancy, OXT appears to facilitate maternal skeletal mobilization for fetal bone ossification through increased osteoclastic resorption and suppressed bone formation (Liu et al., 2009). Genetically modified *Oxt*- and *Oxtr*-deficient mice displayed severe age-related bone loss due mainly to a bone-forming defect (Tamma et al., 2009). Consistent with this, osteoblast- and osteoclast-specific deletion of *Oxtr*s showed low and high bone mass, respectively (Sun et al., 2019).

Vasopressin, a key regulator of serum osmolality and fluid status, has also been implicated in bone remodeling. In contrast to Oxtr-deficient mice, *Avpr*-null mice displayed a high bone mass phenotype arising from increased bone formation and reduced bone resorption, indicating that vasopressin negatively regulates skeletal remodeling (Sun et al., 2016; Tamma et al., 2013). This finding might explain the profound bone loss in patients with chronic hyponatremia, which is often accompanied by high vasopressin levels (Tamma et al., 2013).

Lastly, and importantly, there is emerging evidence that certain pituitary hormones are produced locally by bone marrow-derived cells and regulate bone remodeling in a paracrine manner. ACTH is produced by macrophages (Pállinger and Csaba, 2008), suggesting that MC2R in bone may be regulated locally in addition to its systemic control. Macrophage and CD11β^+^ cells also express a splice variant of TSH, TSHβv, which is biologically active and confers an osteoprotective effect (Baliram et al., 2013; Baliram et al., 2016). In all, therefore, new pituitary-bone circuitry of biologic and medical importance continues to evolve through the use of genetically mouse models. This provides the framework for the extension of such circuitry in the regulation of other somatic and central functions, such as body fat regulation, energy metabolism, inflammation, and central neural functions, by pituitary hormones—a new physiology that is just beginning to be unearthed.

## Two-way traffic between bone and brain

A brain-bone connection has been evident from multiple human and mouse studies. Early studies established the SNS as a negative regulator of bone formation through the action of the adipokine, leptin, and the hypothalamic leptin receptor (LEPR) (Yamashita et al., 1998; Figure 2). Intracerebroventricular leptin administration reduced bone mass and bone formation (Ducy et al., 2000), actions that were mediated by osteoblastic β2-adrenergic receptors (Adrb2) (Takeda et al., 2002). The therapeutic potential of non-selective β-adrenergic antagonists, like propranolol, in bone mass regulation in people has also been confirmed (Reid et al., 2005; Schlienger et al., 2004; Takeda et al., 2002). In contrast, the osteoclastic effect of leptin is facilitated by two distinct, antagonistic pathways. Leptin-mediated SNS activation promotes osteoclastogenesis through increased RANKL expression, which is counteracted by increased secretion of the neuropeptide CART by the hypothalamus secondary to leptin-LEPR binding (Elefteriou et al., 2005).

At the level of sympathetic ganglia, leptin-enhanced sympathetic outflow is initiated by the transcription factor FOXO1, which in turn increases the expression of dopamine β-hydroxylase (Kajimura et al., 2014). The upregulation of molecular clock genes, namely *Per* and *Cry*, downstream of Adrb2 activation promotes osteoblast proliferation through upregulation of *c-fos* and *Jun* (Fu et al., 2005). These findings suggest that the process of bone remodeling relies on oscillations of gene expression or circadian rhythmicity (Fu et al., 2005). Of note, parasympathetic nerve terminals originating from the spinal cord release acetylcholine (ACh) to interact with nicotinic ACh receptors and antagonize SNS tone, thus inhibiting bone resorption and increasing bone mass (Bajayo et al., 2012). Central parasympathetic regulation is mediated by IL-1 (Bajayo et al., 2012; Bajayo et al., 2005).

In addition to SNS and parasympathetic regulation, multiple other neuronal signaling cascades have been implicated in the complex neuronal-bone interaction, namely, melanocortin-4 receptor, Y-receptor, cannabinoid receptor, and neuromedin U (Bajayo et al., 2005; Baldock et al., 2002; Karsak et al., 2005; Ofek et al., 2006; Sato et al., 2007; Shi and Baldock, 2012). The peripheral cannabinoid receptor (CB2) that regulates appetite and energy balance also regulates bone turnover by modulating sympathetic innervation. Mice with a targeted deletion of *Cb2* gene show markedly accelerated age-related trabecular bone loss and cortical expansion with high bone turnover (Ofek et al., 2006) In addition, GWAS showed an association of a single polymorphism and haplotype encompassing *CB2* gene on human chromosome 1p36 (Karsak et al., 2005). On the other hand, the central cannabinoid receptor type 1 (CB1), which is present in sympathetic terminals, interacts with endocannabinoid 2-arachidonoylglycerol to suppress norepinephrine release and prevent Adrb2 activation in bone, resulting in increased bone mass (Tam et al., 2008). Further, upregulating the NO-cGMP-PKG signaling by inhibiting phosphodiesterase (PDE)-5A, which expressed in sympathetic neurons of the locus coeruleus, raphe pallidus, and paraventricular nucleus of the hypothalamus, suppresses bone remodeling (Kim et al., 2020b). In addition, recent studies show that sympathetic nerves that richly innervate the vestibular cells of the inner ear also regulate bone remodeling peripherally. In fact, bilateral vestibular lesions in mice caused peripheral bone loss due to decreased bone formation and increased resorption (Vignaux et al., 2015). This finding may be relevant to the osteoblast dysfunction in elderly patients with osteoporosis, many of whom also have vestibular dysfunction.

Given the extensive central regulation of skeletal homeostasis, it comes as no surprise that the bone can also signal back to the brain to modulate this regulation. Recent findings indicate that bone-derived signals can affect cognitive function and fetal brain development. GPCRs for osteocalcin, namely Gpr158, have been identified in brain (Khrimian et al., 2017; Obri et al., 2018), and uncarboxylated osteocalcin (GluOCN) has been shown to cross the blood-brain barrier to accumulate in specific regions of the brain, primarily the midbrain and brainstem (Oury et al., 2013). Furthermore, osteocalcin-deficient mice demonstrated a behavioral phenotype of passivity (Ducy et al., 1996; Obri et al., 2018) independently of abnormal glucose homeostasis (Pi et al., 2008). In addition to behavioral changes, osteocalcin-deficient mice of both sexes also displayed major deficits in learning and memory (Oury et al., 2013). Further anatomic examination revealed smaller brain sizes—the dentate gyrus of the hippocampus was 30% smaller and the corpus callosum was often missing, both of which are consistent with decreased spatial learning and memory (Oury et al., 2013). At a biochemical level, the midbrain and brainstem of osteocalcin-deficient mice had significantly lower amounts of monoamine neurotransmitters, including dopamine, serotonin, and norepinephrine. There was also significantly higher accumulation of the inhibitory neurotransmitter GABA in the same regions (Oury et al., 2013). These finding are supported by intracerebroventricular infusions of osteocalcin in *Ocn^-/-^* mice that rescued the anxiety and depression phenotypes (Oury et al., 2013). Furthermore, injections of plasma from wild type mice and osmotic pumps delivering osteocalcin rescued defects in cognition and anxiety (Khrimian et al., 2017). Taken together, these findings show that osteocalcin regulates neurotransmitter synthesis and affect behavior.

## Bone is molecularly tied to muscle

Situated close to each other, bone and muscle work as a functional unit, clinically demonstrated by the fact that osteoporosis occurs with sarcopenia (Edwards et al., 2015). Spinal cord injury, which is associated with severe osteoporosis and progressive muscle loss, is a striking example of the functional interdependence of muscle and bone (Bauman et al., 1999). Spinal cord injury-induced bone resorption and bone loss are normalized after electrical stimulation of denervated muscle in rats (Qin et al., 2013), which suggests a non-neural, molecular connection between bone and muscle.

Skeletal muscle is indeed a recognized endocrine organ, secreting numerous cytokines and growth factors, collectively termed myokines (Gomarasca et al., 2020). Irisin, released upon exercise, is a suspected candidate for a non-neural, bone-muscle link. Irisin administered to rodents increased bone mass by enhancing ERK signaling and upregulating expression of the osteoblastogenic genes *Atf4*, *Runx2*, *Osx*, *Lrp5,* and *β-catenin* (Colaianni et al., 2014; Colaianni et al., 2015; Zhang et al., 2017). Surprisingly, irisin did not only increase bone formation by inhibiting sclerostin expression, but also suppressed RANKL-induced osteoclastogenesis (Zhang et al., 2017). In contrast, myostatin, a member of the TGFβ superfamily, regulates osteogenesis negatively. Myostatin deficiency results in an overall increase in bone density, strength, and mineralization (Carnac et al., 2007; Eijken et al., 2007; Elkasrawy and Hamrick, 2010; McPherron et al., 1997). Myostatin is overexpressed in bone of diabetic *Lepr*^db−/−^ mice with a tibial defect. Inhibiting myostatin by direct injection of its antagonist, follistatin into the site of the defect, resulted in improved bone regeneration, osteoblast proliferation and differentiation, and calcification. Taken together, these findings indicate that follistatin exerts a pro-osteogenic effect secondary to myostatin blockade (Amthor et al., 2004; Cash et al., 2009; Wallner et al., 2017). The negative skeletal remodeling induced by myostatin appears to be the result of suppressed WNT signaling. In murine osteocytes, myostatin-induced epigenetic changes through osteocyte-derived microRNA-218 (miR-218) and other exosomes has been shown to increase sclerostin and DKK1 expression, resulting ultimately in suppressed osteoblastogenesis (Li et al., 2016; Qin et al., 2017).

The deletion of bone-specific, muscle-specific, or commonly expressed genes in both bone and muscle cells also provide further insights into the muscle-bone connection. The osteocyte-specific deletion of the MBTPS1 protease increased muscle regeneration by upregulation of *Pax7*, *Myog*, *Myod1*, *Notch,* and *Myh3* expression resulting in increased muscle mass and contractility (Gorski et al., 2016). Further, skeletal muscle-specific deletion of the *Baml1* gene, which encodes a molecular clock transcription factor, impaired muscle function, but caused bone and cartilage defects (Schroder et al., 2015). Likewise, osteocalcin, which is primarily secreted from bone cells, promotes nutrient uptake in myofibers with exercise (Mera et al., 2016a; Mera et al., 2016b). The downregulation of methyltransferase 21C*,* which methylate chaperones in bone and muscle, reduces myogenesis as well as osteocyte survival (Huang et al., 2014). Similarly, ryanodine receptors integrate cytosolic Ca^2+^ signals in both osteoclasts and muscle cells (Zaidi et al., 1989; Zaidi et al., 1992; Zaidi et al., 1995).

## Bone connects to fat and energy homeostasis

Bone and adipose tissue remodeling occur through a complex neuroendocrine circuit that involves the brain, pituitary gland, adipose depots, and the skeleton. As noted above, a non-classical action of FSH was implicated not only in bone remodeling (see above), but also in promoting adipogenicity. The perimenopausal transition, which is accompanied by the early rise of FSH followed by estrogen deficiency, is associated with increased visceral obesity and dysregulated energy homeostasis. Our group has shown that inhibiting FSH signaling both genetically in *Fshr*^+/-^ mice and pharmacologically using an FSH-blocking antibodies in mice dramatically reduces fat in all depots, including bone marrow, and induces thermogenic beige adipose tissue (Gera et al., 2020; Zhu et al., 2012a). This action is exerted through high-affinity FSHRs present on both white and brown adipocytes (Liu et al., 2017).

The interaction of the skeleton with the brain and fat is, in part, mediated by adipokines and the SNS. Leptin- and leptin receptor-deficient mice are phenotypically obese and hypogonadal (Ducy et al., 2000). Adiponectin partially counteracts leptin’s action by decreasing sympathetic tone, which is opposed by its peripheral effect by suppressing osteoblastogenesis directly (Kajimura et al., 2013). Also worth noting is that fatty acids secreted by adipocytes, such as palmitate, exert a lipotoxic effect on osteoblasts and osteocytes and their precursors in the bone marrow (Al Saedi et al., 2019; Gunaratnam et al., 2014). ‘Hunger hormones’ such as peptide Y and ghrelin have also been linked to bone loss in patients after gastric bypass due to a paradoxical increase in bone marrow fat (Kim et al., 2020a).

The role of osteocalcin in glucose homeostasis is also noteworthy. GluOCN binds to the GPR6A to stimulate pancreatic β-cell proliferation and insulin secretion; in turn, insulin favors GluOCN bioactivity (Ferron et al., 2008; Fulzele et al., 2010; Pi et al., 2011; Wei et al., 2014b). Osteoblast-specific insulin receptor-deficient mice showed low levels of GluOCN with reduced bone formation. These mice developed obesity and insulin resistance with aging, which was improved by GluOCN administration (Fulzele et al., 2010). Additionally, delta like-1 (DLK) protein, which is expressed by the pancreas in response to GluOCN and counteracts the stimulatory effect of insulin on osteoblast proliferation (Abdallah et al., 2015). Likewise, leptin-induced SNS activation results in the upregulation of osteotesticular phosphatase, which inhibits osteocalcin activity (Hinoi et al., 2008). Finally, osteocalcin not only works as an insulin secretagogue, but also improves insulin sensitivity. Daily administration of GluOCN in mice increased mitochondrial activity in skeletal tissue, associated with increased energy expenditure (Ferron et al., 2012). Obese mice with insulin resistance after high-fat diet displayed decreased GluOCN levels (Wei et al., 2014a). Observational data in humans is, however, somewhat conflicting due to confounding factors and heterogenous study designs. In type 1 diabetes patients, GluOCN was positively associated with the C-peptide/glucose ratio (Thrailkill et al., 2012); however, osteoporotic patients receiving bisphosphonates, known to suppress bone turnover, did not show a correlation between GluOCN levels and glucose homeostasis parameters, such as fasting glucose or insulin levels (Hong et al., 2013).

Lastly, interest in bone marrow fat has gained significant traction in recent years as aging in both sexes and menopause in women are associated with profound increases in bone marrow fat deposition (Suchacki et al., 2016). Bone marrow adipocytes, interestingly, display a signature of osteogenic precursor markers, such as *Osx*, *Runx2,* and *Lepr*, suggesting a mesenchymal origin, as with osteoblasts (Matic et al., 2016b). Some consider BMSC differentiation into a bone marrow adipocyte as the default, unless it is committed to the osteoblast lineage (Pierce et al., 2019). The expression of PPARγ, CCAAT/enhancer-binding protein α, and secreted frizzled related protein 1 promote BMSC commitment to bone marrow adipocyte differentiation, whereas IGF-1 and adiponectin inhibit adipocyte differentiation (Tencerova and Kassem, 2016). Thus, patients receiving thiazolidinedione develop osteopenia as PPARγ activation stimulates adipogenesis at the expense of osteoblastogenesis (Cawthorn et al., 2014; Lu et al., 2016; Tsuchida et al., 2005). And LepR signaling in BMSC promotes adipogenesis and inhibits osteoblastogenesis in response to diet (Yue et al., 2016). The zinc finger nuclease, ZFP467, also plays a role in determining BMSC fate (Quach et al., 2011). ZFP467-deficient mice demonstrate increased trabecular bone volume and a significant reduction in marrow adipose tissue (Le et al., 2021). The osteoanabolic and anti-adipogenic effects of PTH are, in part, mediated by suppressing *Zfp467* expression (Fan et al., 2017; Le et al., 2021). A recent study has shown that a subpopulation of BMSCs, called marrow adipogenic lineage precursors, express RANKL and regulate osteoclastic bone resorption (Yu et al., 2021).

## Bone talks to other vital organs

Vital organs function in coordination to maintain bodily homeostasis, and this crosstalk between organs is achieved through complex biological communications and feedback mediated through cellular, soluble, and neurohormonal pathways (Armutcu, 2019). For example, fibroblast growth factor (FGF) 23 from osteocytes binds to the FGF receptor 1-Klotho complex in the kidney to promote phosphate excretion (Nakatani et al., 2009; Shimada et al., 2004; Shimada et al., 2001). Clinical observation of decreased bone mass in patients with pathologies of other vital organs has provided additional insight into the integrative nature of skeletal physiology. For example, osteoporosis and osteopenia are prevalent with chronic liver diseases, especially with cholestatic liver disease (Ninkovic et al., 2002). In vitro studies have shown that treatment with unconjugated bilirubin or serum from jaundiced patients significantly reduced viability and differentiation of osteoblast-like cells and primary osteoblasts (Janes et al., 1995; Ruiz-Gaspà et al., 2011), with a significantly increased RANKL/OPG ratio (Ruiz-Gaspà et al., 2011). Taurine, which is primarily synthesized in liver, mediates GH-dependent IGF-1 synthesis and subsequently enhances osteoblast function (Clemens, 2014). Since vitamin B_12_ is required for taurine synthesis, the deletion of gastric intrinsic factor causes low bone mass in mice, which is subsequently rescued by taurine supplementation (Clemens, 2014; Roman-Garcia et al., 2014). Likewise, patients with chronic obstructive pulmonary disease, even when clinically stable, often demonstrate increased inflammatory markers and lower BMD (Liang and Feng, 2012). The association of heart disease and osteoporosis is also worth noting. Secondary hyperparathyroidism can occur in patients with heart failure independent of renal function (Altay et al., 2012). Moreover, upregulation of the renin-angiotensin-aldosterone (RAA) axis in heart failure might promote RANKL expression and osteoclast differentiation (Guan et al., 2011). In all, the skeleton is directly and indirectly intertwined with other vital organs, and new advances in integrative physiology continue to expand the breadth of our understanding of skeletal physiology.

## The impact of aging and sex

Aging-related dysfunction in non-skeletal organ can affect skeletal homeostasis. Decreased organ function and chronic inflammation with aging, which cause changes in hemodynamics, RAA system, and SNS, can disturb bone remodeling (Oishi and Manabe, 2020). The Wnt-related proteins were shown to be downregulated in osteoblasts with aging (Rauner et al., 2008), which was partly mediated by increased endogenous glucocorticoids and oxidized lipid-induced PPARγ activation (Manolagas, 2010). In addition, aging-associated changes in HSCs may cause aggressive osteoclastic bone resorption (Møller et al., 2020).

Sex undoubtedly has a major impact on organ crosstalk. Many factors, including sex-specific genes, genetic imprinting, and sex steroids, are involved in the regulation of key signaling pathways. For example, the difference in the relative levels of calcification and fibrosis in heart valves in male and female may be attributed to differences in BMP/TGFβ signaling (Shah and Rogers, 2018). Notably, estrogen directly induces *Bmp2* and *Bmp6* transcription (Zhou et al., 2003) and stimulates SMAD-2/3 protein degradation (Ito et al., 2010).

## Possible new medicines

Understanding the integrative nature of skeletal physiology has opened up the potential for new therapeutic targets for osteoporosis. Osteo-induction by BMPs has long been utilized to accelerate fracture healing, with recombinant human BMP-2 use approved in bone grafts for treating acute, open tibial shaft fractures (Salazar et al., 2016). Our group recently developed a fully humanized, multipurpose blocking antibody specific to FSHβ targeting both postmenopausal osteoporosis and the accompanying visceral obesity and neurodegeneration (Gera et al., 2020). Upregulating LXR activity has been explored as it suppresses osteoclastogenesis and confers cardioprotection, making LXR agonists a potential dual treatment for osteoporosis and cardiovascular disease (Kleyer et al., 2012; Ma et al., 2017). Osteocalcin, given that it regulates bone remodeling, glucose, and energy homeostasis, and seems to be involved in age-related cognitive decline, is a promising multisystem therapeutic target (Obri et al., 2018).

Due to the common signaling pathways involved in the homeostasis of bone and other organ systems, using existing pharmacotherapies in different applications is also possible. For example, our group showed that nitrogen-containing bisphosphonates can directly inhibit the growth of EGFR-driven cancer cells, making it possible to potentially repurpose them to treat lung, breast, gastrointestinal, head and neck, and other cancers (Stachnik et al., 2014; Yuen et al., 2014). PDE5 inhibitors, a class of commonly used drugs for treating erectile dysfunction and pulmonary hypertension, have shown a combined anabolic and anti-resorptive action in the skeleton (Kim et al., 2020b), highlighting the possibility of targeting the NO-cGMP-PKG pathway in treating osteoporosis (Kim et al., 2020c). Similarly, meclizine, an anti-histamine used for treating vertigo and motion sickness, is being tested for its ability to enhance growth in achondroplasia by inhibiting FGF receptor 3, a negative regulator of endochondral bone growth (Matsushita et al., 2015). Lastly, statins, which block HMG-CoA reductase, promote bone formation in rats (; Zhu et al., 2021). Conversely, the lack of HMG-CoA reductase decreases osteoclast survival, indicating the possibility of repurposing statins for treating bone loss (Luegmayr et al., 2004).

In sum, recent interest in skeletal physiology in the context of intercellular and interorgan communication affords a myriad of translational and clinical possibilities. The complex crosstalk links seemingly divergent processes and systems increasingly intimately, with new therapeutic targets being identified at a rapid rate. As implied above, these discoveries are paving the way for a new clinical paradigm, one that entails using single agent to treat multiple, co-existing diseases.
